# Perception of menopause among women of Sarawak, Malaysia

**DOI:** 10.1186/s12905-021-01230-7

**Published:** 2021-02-22

**Authors:** S. A. R. Syed Alwi, I. B. Brohi, I. Awi

**Affiliations:** 1 Department of Family Medicine, Faculty of Medicine and Health Sciences, Universiti Malaysia Sarawak, 94300 Kota Samarahan, Sarawak, Malaysia; 2 Department of Obstetrics and Gynaecology, Faculty of Medicine and Health Sciences, Universiti Malaysia Sarawak, 94300 Kota Samarahan, Sarawak, Malaysia

**Keywords:** Menopause, Perception, Attitude, Sarawak

## Abstract

**Background:**

Various factors, including menopausal status, educational and social background, culture, and physical and emotional health, may influence women’s perceptions of menopause. This study documents the elements influencing attitudes towards menopause among women in Sarawak, Malaysia.

**Methods:**

A face-to-face interview using a validated questionnaire was conducted with 324 Sarawakian women aged 40–65 to determine the mean age of menopause and perceptions and experiences of menopause among these women.

**Results:**

The mean age ± standard deviation of the women was 51.37 ± 5.91 years. Ninety (27.8%) participants were premenopausal, 124 (38.5%) perimenopausal and 110 (33.7%) postmenopausal. The majority of these women (228; 70.4%) were local indigenous inhabitants of Sarawak. The findings show that 22.5% of the participants agreed that problems during menopause are a natural process. While 21.9% of the participants suggested that menopause should be treated medically, 32.3% argued that natural approaches for menopause symptoms are better than hormonal treatments. Seventy-five per cent of the women agreed that the absence of menses after menopause is a relief; meanwhile, 61.2% stated that menopause causes unpleasant symptoms. Notably, 51.7% were not sure whether women become less sexually attractive after menopause, and 51.1% were uncertain as to whether they feel less of a woman following menopause. Finally, 81.7% of participants were unsure if sexual activity is more enjoyable after menopause, and 71.9% were uncertain whether changes in life during menopause are more stressful. Among the different menopausal stages, the premenopausal group of women were noted to have more positive perceptions of menopause compared to the peri- and postmenopausal women. The study also observed that women with a better educational background generally had more positive perceptions of menopause.

**Conclusions:**

The women’s perceptions of menopause in this study were found to correspond to those in other studies on Asian women. Women with higher levels of education and premenopausal women comparatively expressed more positive opinions regarding menopause. Lastly, most of the women noted that menopausal symptoms are unpleasant, but that the absence of menses after menopause is a relief.

## Background

The advancement of modern medicine and improved health care and health care delivery systems have significantly prolonged human life expectancy rates throughout the world. Indeed, in Malaysia, the life expectancy for women has increased from 70.5 years in 1980 to 78.4 years in 2019 [1].

Studies on Malaysian [2–4], Asian [5, 6] and European women [7–11] have indicated that the mean age of menopause is between 49.4 years and 51.1 years, with an average mean of 50.7 years; this implies that a considerable proportion of Malaysian women live one-third of their lives beyond menopause.

Menopause is a natural physiological process in a woman’s life. It results from a lack of oestrogen secretion from ovarian follicles, leading to the absence of menstruation [12, 13]. A woman is labelled as ‘postmenopausal’ when she misses her periods for 12 months consecutively [14, 15]. Due to oestrogen deficiency, women develop climacteric symptoms, including vasomotor, physical, and psychological complications and sexual dysfunction symptoms, and become physically unwell [15–17].

Women’s perceptions of menopause are influenced by various factors, including their cultural, social and educational background and emotional and physical health [12, 16]. Existing studies have found that sociocultural factors can affect women’s attitude, perceptions and experiences towards menopause [7, 8, 13, 17, 18]. In societies where menopause is viewed positively, symptoms are less common. Numerous studies of Western and Asian women, including Japanese and Chinese women, have documented the cultural aspects of menopause [7, 8, 18–27]. Scholars have indicated that the stigma related to menopause begins in early life due to little knowledge and education besides these cultural and social factors [28–31]. Understanding this crucial stage of life may change women’s negative attitudes towards menopause, which cause apprehension and adverse emotional states in women dealing with this condition. Changing women’s views about menopause by empowering them with knowledge may, therefore, cause less emotional distress among them.

There is a paucity of research concerning Malaysian women’s perceptions of menopause, and no such studies have been conducted among women in Sarawak, Malaysia. This study thus aimed to address this research gap by exploring Sarawakian women’s attitudes towards menopause.

## Methods

### Subjects and setting

This cross-sectional study was conducted from December 2017 to May 2018 after approval from the Research and Ethics Committee, Faculty of Medicine, Universiti Malaysia Sarawak. All research methods were performed in accordance with the relevant guidelines and regulations of the Declaration of Helsinki. The study was carried out among women aged between 40 and 65 who visited four women’s health camps, a health campaign launched by a collaboration of local women’s NGOs, around Kuching, Bau, Serian and Semantan in Sarawak, Malaysia. The participants were from various demographic backgrounds; the women were either from rural, semi-urban or urban areas and held different levels of educational experience ranging from no formal education to tertiary-level education.

All women who fulfilled the inclusion criteria were invited to participate in the study. The inclusion criteria consisted of women between the ages of 40 and 65 who had given written consent. Those who were pregnant, breast-feeding, had uncontrolled medical conditions such as hypertension, diabetes mellitus or heart diseases or any other chronic diseases, were undergoing treatment for cancer or were in remission, or had a history of drug or alcohol abuse, were excluded from the study. Those undertaking hormone replacement therapy were also excluded.

### Assessment instrument

A semi-structured questionnaire was used for this study. The questionnaire was divided into three sections, detailed as follows:

1. The socio-demographic data of the women, which included age, marital status, age of marriage, age of menarche, race, religion, educational level, occupation and average household income.
2. The women’s menopausal status, which was classified according to the Stages of Reproductive Aging Workshop (STRAW) categorisation [15]. STRAW divides menopause staging into *postmenopausal* (no menstrual bleeding in the previous 12 months); *late perimenopause* (menstruation in the last 12 months but not in the last two months; *early perimenopause* (increasing irregularity of menses without skipping periods; seven days’ difference from the beginning of a given cycle to the next, experienced after the previous regular cycle) and *premenopause* (minor changes in cycle length, particularly a decreasing cycle length). The *early* and *late perimenopausal* transition stages were combined and classified as the *perimenopausal stage* to aid statistical analysis [14].
3. The women’s perceptions of menopause, measured using a questionnaire based on the Menopause Attitudes Scale questionnaires constructed by Lieblum and Swartzman [32], local beliefs, and a focus group interview comprising nine, randomly selected menopausal women.

With the help of experienced health workers and language experts, the questionnaire was then created and reviewed by two gynaecologists and a public health professor to ascertain its validity. It consists of 10 items and initially used a 5-point scale (where 1 = strongly agree, 2 = agree, 3 = not sure, 4 = disagree, 5 = strongly disagree). However, after a pilot study of 30 women, nurses and lecturers to validate the constructed questionnaires, some modifications were made to the grading method of each item due to difficulties faced by the women to grade their agreement on the scale. Thus, the scale was reduced to three items: ‘agree’, ‘disagree’ and ‘not sure’. Factor analysis was performed on the modified questionnaire with Cronbach’s alpha ranging from 0.803 to 0.832, indicating that the questionnaire had good reliability.

Interviews were conducted face-to-face in the Malay language; the national language is widely spoken in Malaysia by trained health personnel, and its use was crucial in helping participants to understand questions and give correct answers. During registration, participants who fulfilled the criteria were invited to participate in the study. Explanations were given and written informed consent was obtained. A total of 386 women were invited to participate.

### Statistical analysis

The Statistical Package for the Social Sciences Software Version 25.0 (SPSS, Chicago, IL) was used for analyses. Data were presented as mean ± standard deviations (SD), median and percentages. The *X*^2^ test was applied to compare the categorical data. The *p* value of < 0.05 was considered statistically significant.

## Results

Three hundred and eighty-six women aged 40–65 were invited to participate in this study. Sixty-two participants either did not provide consent, had medical problems, or did not complete the interviews or questionnaire; thus, 324 women completed the study.

The mean age ± SD of the women was 51.37 ± 5.91 years. The mean age at menopause for postmenopausal women was 52.34 ± 3.21 years, while the mean age of menarche among all the women was 11.71 ± 2.14 years, and the mean age of marriage was 19.24 ± 2.27 years. Among the women, 90 (27.8%) were premenopausal, 124 (38.5%) perimenopausal and 110 (33.7%) postmenopausal. Most of the women (228; 70.4%) were local indigenous women of Sarawak; indigenous groups of people in Sarawak include the Iban, Bidayuh, Malay, Melanau, Lumbawang, Kelabit, Kayan and Penan peoples. One hundred and sixty-six participants were Christians (51.23%), and 232 were married (71.60%). The majority (282; 87.03%) had received a formal education; 155 (47.83%) were housewives and 122 (37.4%) had a monthly household income below RM 1,000 (USD 238; 1 USD = RM 4.2; see Table 1).

**Table 1:** Socidemographic characteristics of the study population (n = 324)

Sociodemographic data	*n*	%
Mean age ± SD of the women 51.37 ± 5.91		
Mean age by age group (years)		
42.06 (40–44)	58	17.90
46.83 (45–49)	95	29.32
52.12 (50–54)	78	24.07
56.94 (55–59)	60	18.51
62.01 (60–65)	33	10.18
Ethnic distribution		
Iban	90	27.77
Chinese	96	29.62
Bidayuh	60	18.52
Malay	50	15.43
Melanau	18	5.55
Others (Lumbawang,Kelabit, Kayan, Penan)	10	3.10
Religion		
Christian	166	51.23
Muslim	70	21.60
Buddhist	68	20.98
Others	20	6.17
Marital status		
Married	232	71.60
Widow/divorcee	88	27.16
Single	4	1.23
Educational level		
No formal education	42	12.98
Primary level	102	31.50
Secondary level	140	43.20
Tertiary level	40	12.40
Occupation		
Housewife	155	47.83
General worker	40	12.34
Semi-professional	100	30.86
Professional	29	8.95
Household income (monthly)		
Below RM 1000	122	37.40
RM 1001–RM 2000	54	16.80
RM 2001–RM 3000	65	19.90
RM 3001–RM 4000	35	10.90
RM 4001–RM 5000	18	5.70
Above RM 5000	30	9.30

Table 2 presents the women’s perceptions and attitudes towards menopause. As can be seen, 22.5% of the participants agreed that problems in menopause are a natural process. Approximately 21.9% argued that menopause should be treated medically, while 32.3% suggested that natural approaches are preferable to hormonal treatments for menopause symptoms. The majority of the women (75.0%) agreed that the absence of menses after menopause is a relief; nonetheless, 61.2% indicated that menopause causes unpleasant symptoms. Perhaps surprisingly, only 28.1% of the respondents agreed that they look forward to not worrying about pregnancy after menopause. Meanwhile, 51.7% of the women expressed uncertainty regarding whether women become less sexually attractive after menopause, and 51.1% were unsure if they feel less of a woman following menopause. Lastly, 81.7% of the women were not sure if sexual activity is more enjoyable after menopause, and 71.9% were uncertain as to whether changes in life during menopause are more stressful. Among the menopausal stages, the premenopausal group of women were noted to have the most positive perceptions followed by perimenopausal and postmenopausal women (Table 3). In this study, women with higher educational backgrounds (particularly those with a tertiary-level education) exhibited a more positive attitude towards menopause (Table 4).

**Table 2:** Positive and negative perceptions towards menopause among Sarawak women (*n* = 324)

	Agree (n) (%)	Disagree (n) (%)	Not sure (n) (%)
Positive perceptions			
Menopause is a natural process	73 (22.5)	44 (13.5)	207 (64.0)
Natural approaches for menopausal symptoms are better than hormonal	105 (32.3)	18 (5.6)	201 (62.1)
Absence of menses after menopause is a relief	243 (75.0)	2 (0.6)	79 (24.4)
After menopause, no changes in sex enjoyment	9 (2.8)	50 (15.4)	265 (81.7)
Not to be worried about pregnancy	91 (28.1)	7 (2.2)	226 (69.7)
Negative perceptions			
Menopause should be treated medically	71 (21.9)	29 ( 9.0)	224 (69.1)
Women are less sexually attractive after the menopause	113 (34.8)	44 (13.5)	167 (51.7)
Feels less of a woman following menopause	79 (24.2)	80 (24.7)	165 (51.1)
Menopause causes unpleasant symptoms	198 (61.2)	0 (0%)	126 (38.8)
Changes in life during menopause are stressful	89 (27.5)	2 (0.6)	232 (71.9)

**Table 3:** Positive and Negative Perceptions towards menopause according to menopausal status among Sarawak women (N = 324)

Perception of menopause	Premenopausal (n = 90; 27.8%) Perimenopausal (n = 124; 38.5%) Postmenopausal (n = 110; 33.7%)									*p* value
	Agreen (%)	Disagree n (%)	Not Sure n (%)	Agree n (%)	Disagree n (%)	Not Sure n (%)	Agree n (%)	Disagree n (%)	Not Sure n (%)	
Positive perceptions										
Menopause is a natural process	43 (47.7)	15 (16.6)	32 (35.5)	18 (14.5) *	11 (8.8)	95 (76.6)	12 (10.9)	18 (16.3)	80 (72.7)	< 0.05
Natural approaches for menopausal symptoms are better than hormonal	28 (31.1)	4 (4.4)	58 (64.4)	39 (31.4) §	6 (4.8)	79 (63.7)	38 (34.5)	8 (7.2)	64 (58.1)	< 0.05
Absence of menses after menopause is a relief	69 (76.6)	1 (1.1)	20 (22.2)	70 (56.4)	0 (0)	54 (43.5)	104 (94.5) †	1 (0.9)	5 (4.5)	< 0.05
After menopause, no changes in sex enjoyment	4 (4.4)	18 (20)	68 (75.5)	2 (1.6)	15 (12)	107 (86.2)	3 (2.7)	17 (15.4)	90 (81.8)	
Not to be worried about pregnancy	37 (41.1)	4 (4.4)	49 (54.4)	23 (18.5)**	1 (0.8)	100 (80.6)	31 (28.1)**	2 (1.8)	77 (70)	< 0.05
Negative perceptions										
Menopause should be treated medically	29 (32.2)	12 (13.3)	49 (54.4)	24 (19.3)	8 (6.4)	92 (74.1)	18 (16.3)	9 (8.1)	8375.4)	
Women are less sexually attractive after the menopause	42 (54.4)	17 (18.8)	31 (34.4)	31 (25)	11 (8.8)	82 (66.1)	40 (36.3)	16 (14.5)	54 (49)	
Feels less of a woman following menopause	32 (35.5)	26 (28.8)	32 (35.5)	19 (15.3) **	31 (25)	74 (59.6)	28 (25.4) **	23 (20.9)	59 (53.6)	< 0.05
Menopause causes unpleasant symptoms	78 (86.8)	0 (0)	12 (13.3)	69 (55.6)	0 (0)	55 (44.3)	51 (46.3)	0 (0)	59 (53.6)	
Changes in life during menopause are stressful	29 (32.2)	2 (2.2)	59 (65.5)	38 (30.6)	0 (0)	86 (69.3)	22 (20)	1 (0.9)	87 (79)	

*Significant difference *p* < 0.05 compared to postmenopausal in agreeing with the statement

^§^Significant difference *p* < 0.05 compared to pre- and post-menopausal in agreeing with the statement

^†^Significant difference *p* < 0.05 compared to pre- and peri-menopausal in agreeing with the statement

**Significant difference *p* < 0.05 compared to premenopausal in agreeing with the statement

**Table 4:** Positive and negative perceptions towards menopause according to educational level among Sarawak women (N = 324)

Perception of menopause	No formal education (n = 42)			Primary level (n = 102)			Secondary level (n = 140)			Tertiary level (n = 40)			*p* value
	Agree n (%)	Disagree n (%)	Not SURE n (%)	Agree n (%)	Disagree n (%)	Not Sure n (%)	Agree n (%)	Disagree n (%)	Not sure n (%)	Agree n (%)	Disagree n (%)	Not sure n (%)	
Positive perceptions													
Menopause is a natural process	6 (14.2)	5 (11.9)	31 (73.8)	20 (19.6)	15 (14.7)	67 (65.6)	29 (20.7)	14 (10)	97 (69.2)	18 (45)^†^	10 (25)	12 (30)	< 0.05
Natural approaches for menopausal symptoms are better than hormonal	11 (26.1)	3 (7.1)	28 (66.6)	28 (27.4)	5 (4.9)	69 (67.6)	40 (28.5) *	7 (5)	93 (66.4)	26 (65)^†^	3 (7.5)	11 (27.5)	< 0.05
Absence of menses after menopause is a relief	38 (90.4)	0 (0)	4 (9.5)	78 (76.4)	1 (0.9)	23 (22.5)	89 (63.5) *	1 (0.7)	50 (35.7)	38 (95)^†^	0 (0)	2 (5)	< 0.05
After menopause, no changes in sex enjoyment	1 (2.3)	5 (11.9)	36 (85.7)	2 (1.9)	7 (6.8)	93 (91.7)	4 (2.8)	18 (12.8)	118 (84.2)	2 (5)	20 (50)	18 (45)	
Not to be worried about pregnancy	4 (9.5)	1 (2.3)	37 (88)	9 (8.8)	1 (0.9)	92 (90.1)	41 (29.2)	3 (2.1)	96 (68.5)	37 (92.5)^†^	2 (5)	1 (2.5)	< 0.05
Negative perceptions													
Menopause should be treated medically	3 (7.1)	4 (9.5)	35 (83.3)	19 (18.6)	6 (5.8)	77 (75.4)	33 (23.5) *	9 (6.4)	98 (70)	16 (40)	10 (25)	14 (35)	
Women are less sexually attractive after the menopause	8 (19)	7 (16.6)	27 (64.2)	18 (17.6)	9 (8.8)	75 (73.5)	59 (42.1) *	17 (12.1)	64 (45.7)	28 (70)^†^	11 (27.5)	1 (2.5)	< 0.05
Feels less of a woman following menopause	11 (26.1)	12 (28.5)	19 (45.2)	21 (20.5)^††^	22 (21.5)	59 (57.8)	30 (21.4)	27 (19.2)*	83 (59.2)	17 (42.5)	19 (47.5)	4 (10)	< 0.05
Menopause causes unpleasant symptoms	14 (33.3)	0 (0)	28 (66.6)	43 (42.1)	0 (0)	59 (57.8)	108 (77.1)	0 (0)	32 (22.8)	33 (82.5)^†^	0 (0)	7 (17.5)	< 0.05
Changes in life during menopause are stressful	12 (28.5)	0 (0)	30 (71.4)	32 (31.3)	1 (0.9)	69 (67.6)	30 (21.4)	1 (0.7)	109 (77.8)	15 (37.5)	1 (2.5)	24 (60)	

*Significant difference *p* < 0.05 compared to no formal education and primary level in agreeing with the statement

^†^Significant difference *p* < 0.05 compared to no formal education, primary level and secondary level in agreeing with the statement

^††^Significant difference *p* < 0.05 compared to no formal education in agreeing with the statement

## Discussion

Various factors determine a woman’s perceptions of menopause. Indeed, menopausal status, social background, education, occupation, physical or emotional health, and general symptoms may influence women’s attitudes towards menopause [18, 19]. While menopause has been extensively investigated elsewhere, studies concerning menopause are limited in Malaysia.

Many studies focusing on both Asian women and women globally have reported mixed responses regarding women’s perceptions of menopause and how they, their society, or their communities view menopause. Similar observations were found in this study: more than 50% of the women interviewed were unable to measure or were not sure of their perceptions on menopause [2, 20–26].

In his study, Ismail [2] suggested that 16% of women regard menopause as a ‘pity’; meanwhile, 60% were pleased, and 24% felt it did not matter. NorHayati [20] found that 73.1% of women did not view menopause as a medical condition, while 71.7% did not feel less of a woman after menopause.

A study conducted in Thailand by Chirawathul et al. [21] observed that rural women regarded menopause as a simple and natural biological event which requires no treatment; they also expressed relief that they no longer need to worry about pregnancy after menopause. Mazhar et al. [22] suggested that Pakistani women have different views about menopause. According to the authors, few participants saw menopause as a medical condition requiring treatment; the majority considered it a natural transition. In contrast, most of the women (69.1%) in the present study were not sure as to whether menopause is a medical problem or not.

In recent years, two community studies in Taiwan [23, 24] indicated that Taiwanese women were highly aware of menopause (97%), with a very positive and accepting attitude towards menopause. They also demonstrated a considerable willingness to receive medical treatment. Another study by Leon et al. [25] focused on middle-age Ecuadorian women who exhibited positive attitudes towards menopause: 93.7% viewed it as a normal event and not a problem; 65.3% expressed relief at the diminished risk of pregnancy, and 60.7% reported that life is more straightforward and calmer.

Finally, Sinclair et al. [26] found that 80% of women agreed with the view that because reduced hormone levels bring about the menopause, it should be viewed as a medical condition and treated as such. The majority of these participants (76.2%) also disagreed that a woman feels less of a woman after menopause. Conversely, in the present study, only 22.5% agreed menopause is a natural process; 21.9% argued that menopause should be treated medically, and just 24.2% of the respondents feel like less of a woman following menopause.

This study found that most of the women (69.1%) were uncertain whether to treat menopause medically, while 62.1% were unsure if a more natural approach, using traditional, herbal or alternative medicine, was better than a hormonal method.

In contrast, in a study among Kelantanese women in Malaysia [20], NorHayati noted that 73.1% of her respondents did not view menopause as a medical condition. Similarly, Chirawathul et al. [21] also suggested that Thai women regarded menopause as a simple and natural biological event which requires no treatment. Mazhar et al. [22], in their study concerning Pakistani women, reported that few respondents viewed menopause as a medical condition requiring treatment; rather, the majority considered it a natural transition. In Larroy et al.’s [33] study in an Asian context, menopause is treated as a natural process of ageing and associated with higher social status and wisdom among the communities. Conversely, a study by Sinclair et al. [26] found that the majority of their respondents agreed with the view that because diminished hormone levels bring on the menopause, it should be viewed as a medical condition and treated as such. In two Taiwanese community studies [23, 24], meanwhile, women were highly aware of menopause (97%), exhibiting a positive, accepting attitude and great willingness to receive medical treatment.

Most of the respondents in the present study (75.0%) agreed that the absence of menses after menopause is a relief, although 61.2% noted that menopause causes unpleasant symptoms. However, only 28.1% agreed that they look forward to not worrying about pregnancy after menopause. These findings correspond with Leon et al.’s study [27] focusing on middle-age Ecuadorian women. The researchers observed positive attitudes towards menopause, where 93.7% of respondents viewed it as a normal, unproblematic event; 65.3% expressed relief at the diminished risk of pregnancy, and 60.7% reported that life is easier and calmer. Another study by Chirawathul et al. [21] noted that Thai women felt better after menopause as they no longer have to worry about pregnancy. Finally, Wong et al. [27] found that their participants, consisting of middle-aged women in Kuala Lumpur, held positive attitudes towards menopause. While most agreed that menopause indicates loss of youth and fertility and a sign of ageing, others argued that menopause is simply a part of growing old and involves freedom from menstruation, pregnancy and childbirth. Negative attitudes towards menopause, such as treating it as a disease, no longer feeling like a real woman or feeling old and useless, and the loss of drive to perform daily chores were rejected by most participants. Surprisingly, however, despite the prevalence of positive attitudes towards menopause, many participants also expressed nervousness, fear and sadness about approaching menopause [27].

One could conclude, from this study’s findings, that the majority of the respondents refuted the idea that menopause and sexual activity are related. Indeed, only 2.8% of the women agreed that sex is more enjoyable after menopause; meanwhile, 34.8% argued that women are less sexually attractive after menopause and 24.2% feel like less like a woman after menopause.

When comparing the status or stages of menopause, those with positive perceptions of menopause in this study were generally among the premenopausal group. The same findings were noted by Larroy et al. [33], Wong et al. [27] and several other studies focusing on Asian and European women, where positive perceptions were shared more by younger women than older women in their postmenopausal stages [20, 21, 23, 25].

Levels of education and background were also positive influences among women regarding menopause: the present study, along with several others, found that women with a stronger educational background generally have a better and more positive perception of menopause [6, 14, 28–33].

Literacy and knowledge concerning menopause can be increased by various methods, such as arranging health talks in the community and using social media platform. Malaysia, and especially Sarawak, has a mixed population of urban and rural. In remote areas where internet access is sporadic, community participation and engagement through initiatives like health camps play a prominent role in educating women regarding menopause. While it was challenging to transfer knowledge about menopause to women with low education levels, it was inspiring to share knowledge among more highly educated women, particularly in an urban setting, where the potential of social media can be utilised at its fullest in educating not only women but also the general population.

While collecting data, some participants appeared to express reservations about sensitive questions, especially those regarding sex. This could be due to the norms of Malaysian and Asian society, whereby women are reluctant to openly discuss this topic. Another limitation was the difficulty in translating the question into different local dialects/languages; however, in order to minimise this effect, the researcher conducted face-to-face interviews with the help of a translator and explanation was given to respondents who had problems understanding the questions.

## Conclusions

The study of the perception of menopause among Sarawak women between the ages of 40 and 65 showed the mean age of menopause was 52.34 ± 3.21 years. Participants’ attitudes towards menopause differ from those reported in other studies based in Malaysia and other Asian countries. This discrepancy could be because of misunderstandings or misconceptions regarding menopause among Sarawak women. Educational background has a significant effect on how women perceive menopause: the higher the educational level, the more positive the perception of menopause. Moreover, since menopause is an issue which is not generally discussed openly in most eastern communities, including among Sarawak women, there seems to be lack of information, knowledge and awareness regarding menopause; this was reflected in the answers given during the interviews.

Primary care providers, including primary care physicians, play an essential role in educating and creating awareness among women of all ages about menopause since primary care providers are the first point of contact with formal health care providers for individuals, families and communities.

## Data Availability

The data used and/or analyzed during the current study are available from the corresponding author upon request.
